# Hindcasting of nutrient loadings from its catchment on a highly valuable coastal lagoon: the example of the Fleet, Dorset, UK, 1866–2004

**DOI:** 10.1186/1746-1448-2-15

**Published:** 2006-12-29

**Authors:** Geraint J Weber, Patrick E O'Sullivan, Paul Brassley

**Affiliations:** 1 Environment Agency Wales, Cambria House, 29 Newport Road, Cardiff, CF24 0TP, UK; 2 School of Earth, Ocean and Environmental Sciences, University of Plymouth, Drake Circus, Plymouth PL4 8AA, UK; 3 School of Geography, University of Plymouth, Drake Circus, Plymouth PL4 8AA, UK

## Abstract

**Background:**

Nutrient loadings from its catchment upon The Fleet, a highly valuable coastal lagoon in Southern England, were hindcast for the period AD 1866–2004, using a catchment model, export coefficients, and historical data on land use changes, livestock numbers, and human population. Agriculture was the main nutrient source throughout, other inputs representing minor contributions. Permanent pasture was historically the main land use, with temporary grassland and cereals increasing during the mid-20th century. Sheep, the main 19th century livestock, were replaced by cattle during the 1930s.

**Results:**

Total nitrogen loadings rose from *ca *41 t yr-1 during the late 19th century to 49–54 t yr-1 for the mid-20th, increasing to 98 t yr-1 by 1986. Current values are *ca *77 t yr-1. Total phosphorus loads increased from *ca *0.75 t yr-1 for the late 19th century to *ca *1.6 t yr-1 for the mid-20th, reached *ca *2.2 t yr-1 in 1986, and are now *ca *1.5 t yr-1. Loadings rose most rapidly between 1946 and 1988, owing to increased use of inorganic fertilisers, and rising sheep and cattle numbers. Livestock were the main nutrient source throughout, but inputs from inorganic fertilisers increased after 1946, peaking in 1986. Sewage treatment works and other sources contribute little nitrogen, but *ca *35% of total phosphorus. Abbotsbury Swannery, an ancient Mute Swan community, provides *ca *0.5% of total nitrogen, and *ca *5% of total phosphorus inputs.

**Conclusion:**

The Fleet has been grossly overloaded with nitrogen since 1866, climaxing during the 1980s. Total phosphorus inputs lay below 'permissible' limits until the 1980s, exceeding them in inner, less tidal parts of the lagoon, during the 1940s. Loadings on Abbotsbury Bay exceeded 'permissible' limits by the 1860s, becoming 'dangerous' during the mid-20th century. Phosphorus stripping at point sources will not significantly reduce loadings to all parts of the lagoon. Installation of 5 m buffer strips throughout the catchment and shoreline will marginally affect nitrogen loadings, but will reduce phosphorus inputs to the West Fleet below 'permissible' limits. Only a combination of measures will significantly affect Abbotsbury Bay, where, without effluent diversion, loadings will remain beyond 'permissible'.

## Background

Eutrophication is a widespread and pernicious environmental problem, especially when it affects valuable sites of great ecological value and importance, where it may incur rehabilitation costs measured in millions of dollars per year [1]. In order to reverse the process, it is first necessary to identify the relevant nutrient sources [2], and then to reconstruct their respective impact over time. Such knowledge may then be used in order to develop and to evaluate realistic and sustainable alleviation and/or restoration strategies.

Information is acquired using three main approaches, namely

1. palaeolimnological reconstruction of changing nutrient concentrations and/or loads using microfossil transfer functions [3].

2. 'hindcasting' of present nutrient loadings based on export coefficient calculations using historical records [4-6].

3. a combination of the two [7,8].

The data obtained are then used to reconstruct the timing of respective episodes of nutrient enrichment (and hence their cause), and to evaluate the likely effects of alternative rehabilitation strategies [9]. Here we present a reconstruction of nitrogen and phosphorus loadings on the Fleet, a highly valuable eutrophic coastal lagoon in Southern England, developed using the hindcasting approach, for the period AD 1866–2004. These results are then used to evaluate possible restoration strategies, including removal of point discharges, and installation of riparian buffer strips.

### Site description

**Figure 1:**
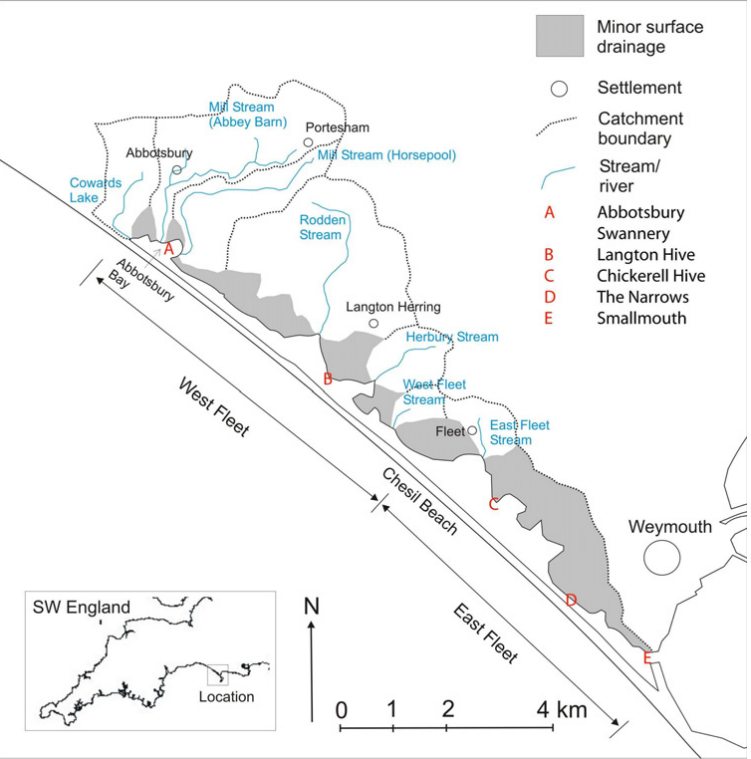
The Fleet Lagoon and its catchment.

The Fleet (Figure 1, Table 1) is a saline lagoon impounded behind Chesil Beach, a natural coastal shingle barrier extending *ca *13 km from Portland to Abbotsbury, Southern England [10], and the finest UK example of its kind [11]. In terms of its physical characteristics and biodiversity, the Fleet is also probably one of the most important coastal lagoons in Atlantic Europe, containing a large range of habitats and species, a wide variety of common and unusual lagoonal specialists (some of which are national rarities), and a high degree of 'naturalness' [12,13].

**Table 1:** The Fleet: morphometric and other data (from [19], [29], [31]).

Latitude	50° 35' N	
Longitude	2° 30' W	
Area (A, ha)	480	
Length (L, km)	13	
	Minimum	Maximum

Width (m)	65	900
Depth (Z, m)	0.5	5.2
Catchment area (D, km^2^)	28.07	

Tidal waters enter the Fleet *via *Portland Harbour (Figure 1), and a narrow channel at Smallmouth at the southeast end of the lagoon, with a maximum phase lag of 5.25 hours at high water springs, and 8–12 hours at low water [14,15]. Hydrography generates a complex tidal and salinity regime, producing an ecologically important contrast between the strongly tidal and essentially (near) marine East Fleet, and the more brackish West Fleet, where there is a still strong salinity gradient, but a much smaller tidal range [16,17]. The lowest salinities are observed in Abbotsbury Bay, at the extreme northwest end of the lagoon. Seawater also enters the Fleet *via *the Chesil Beach, but this process (*pace *[18]) represents only a minor input [14,19].

Nutrient concentrations [12,20] are highest in Abbotsbury Bay and the West Fleet, falling to much lower values close to the entrance to the lagoon (Table 2). The impact of catchment nutrient loadings is therefore greatest in the innermost parts of the lagoon, where reduced tidal flushing, high temperatures and high pH together produce extreme conditions. Here, macro- and microalgal blooms and oxygen deficits [20], the greatest concentrations of chlorophyll *a*, and the highest ratios of nitrogen to phosphorus [12] are observed, and filamentous green algae have at least partly replaced *Zostera *and *Ruppia *[21]. In 2002, the Fleet was designated as a Polluted Water (eutrophic) under the EC Nitrates Directive (91/271/EEC), and its catchment a Nitrate Vulnerable Zone (NVZ), a measure designed to reduce losses of nitrogen to the lagoon, principally from agriculture [20].

**Table 2:** Mean annual nutrient concentrations, N:P ratios and chlorophyll a concentrations for the Fleet Lagoon (from [12]).

	TON (mg l^-1^)	Orthophosphate (μg l^-1^)	N:P ratio (TON: PO_4_^-^)	Chlorophyll *a *(μg l^-1^)
***West Fleet***				
Abbotsbury Swannery	1.96	135.67	14.56	58.83
Langton Hive	0.46	30.67	15.89	9.50
***East Fleet***				
Chickerell Hive	0.32	21.00	15.69	8.49
The Narrows	0.19	16.43	12.06	5.40
Smallmouth	0.09	9.75	10.34	2.94

N = TON plus ammonia

The bed of the Fleet is occupied seasonally by macrophytic seagrasses (*Zostera *spp.), tassleweeds (*Ruppia *spp.), and populations of the rare macroalga, the Foxtail Stonewort (*Lamprothamnium papulosum *(Wallr.) J. Groves; [22]). In Abbotsbury Bay, macrophytes are replaced by green algae ('silk weed'; [23,24]), and in other bays by sea lettuce (*Ulva lactuca*; [25]). Other rarities are present in the macrozoobenthos. Fish populations contain a mixture of marine and freshwater taxa, and the Fleet is protected as a summer nursery for juvenile marine species.

Fleet Lagoon and Chesil Beach Nature Reserve was established during the 1970s. The Fleet is also a UK Grade 1 SSSI, a Ramsar wetland of world importance, a Priority Habitat under the EC Habitats Directive, (92/43/EEC), a Special (EU) Protection Area (SPA) under the EC Directive on Conservation of Wild Birds (79/409/EEC) [20], and an SAC included in the Marine Special Areas of Conservation (Marine SACs) Project, part of the UK response to the European Water Framework Directive (2000/60/EC; [26]). It was also recently incorporated in the Dorset and East Devon Coast World Heritage Site [27]. Threats to its unique ecological character broadly include influx of exotic species, changes in hydrological regime, tourism, military activities, and eutrophication [12,20,26].

Abbotsbury Swannery, located at the northwest end of the lagoon, is one of the largest UK colonies of Mute Swans (*Cygnus olor *L.), and has existed for at least seven hundred years [28]. Since 1970, maximum numbers have risen from *ca *50 to *ca *400 adult birds [29], and concerns have been expressed that the Swannery constitutes a significant nutrient source to the lagoon. Total wildfowl counts (not just Mute Swans) for the Fleet (1983–1993) are given by Fair [30].

### The catchment of the Fleet

The catchment of the Fleet (Figure 1) is underlain by Jurassic limestones and clays, producing soils of high quality, most of which have been under continuous cultivation for more than 3000 years [28]. The majority of surface drainage (71%), calculated using GIS [31], is to the West Fleet. Of this, 38% enters *via *Abbotsbury Bay. Apart from East Fleet Stream (4%), the East Fleet is served by direct drainage only. Land use is predominantly agricultural, with only *ca *215 ha of non-agricultural land. Permanent grassland currently makes up 40% of agricultural land, cereals *ca *27%, and temporary grassland 14% [32].

The main point sources in the catchment of the Fleet are Abbotsbury and Langton Herring STWs, both of which discharge into the West Fleet [33]. Consent to discharge (<*ca 50 *m^3 ^day^-1^) is also granted to a number of small local businesses and residences. Besides agriculture, other diffuse ('non-point') sources comprise wildfowl (including Abbotsbury Swannery), Portland Harbour, groundwater and saline intrusions, and aerial deposition.

Estimated current annual loadings from catchment sources (total = *ca *130 t N, 3.3–4.1 t P yr-1), derived using export coefficients [33], are listed in Table 3. For nitrogen, *ca *109 t yr-1 (84%) are contributed by agriculture, and only *ca *1.5–3 t yr-1 (1–2.5%) by point sources. For phosphorus, the relevant values are *ca *2.3 t yr-1 (55–70%) from agriculture, and *ca *0.5–1.3 t yr-1 (12–39%) from STWs. Of these, *ca *86% of the total nitrogen loading (106 t), and *ca *68% of phosphorus (2.1 t), enter *via *streams draining into the West Fleet, and *ca *37.5% of total nitrogen loading (46 t), and *ca *53% of phosphorus (1.7 t) into Abbotsbury Bay.

**Table 3:** Estimated annual nutrient loadings on the Fleet (from [33]).

Source	Nitrogen		Phosphorus	
	
	t yr-1	%	t yr-1	%
Point sources (STWs)	1.5 – 3.2	1 – 2.5	0.48 – 1.29	12 – 39
Livestock	44.4	34	1.54	37 – 47
Synthetic Fertilisers	64.4	49 – 50	0.73	18 – 23
Background	18.5	14	0.42	10 – 13
Abbotsbury Swannery	0.3	0.2	0.06	2
Other bird species	0.2	0.1	0.06	2
Total	129.3–131.0		3.29–4.10	

## Results

Nutrient loadings upon the Fleet from its catchment were hindcast for the period AD 1866–2003 using

1. historical records of local land use change and livestock numbers stored at the Public Record Office, Kew, London,

2. export coefficients for livestock, land use types and fertiliser use derived from previous literature, and

3. a catchment model which sums loads from all relevant sources.

Further details are given in the Methods section below.

### Agricultural land use

**Figure 2:**
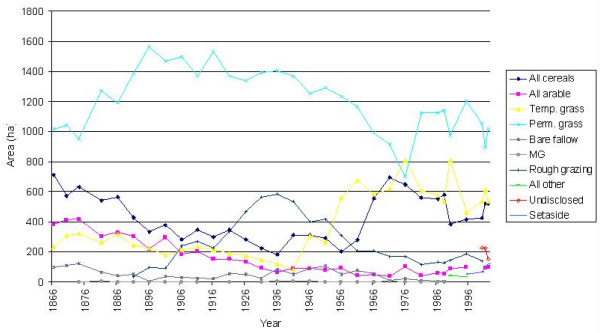
Land use changes (ha) in the catchment of the Fleet Lagoon, AD 1866–2202.

Since 1866, the main agricultural land use in the catchment of the Fleet has been permanent pasture (Figure 2). Permanent grassland initially accounted for *ca *42% of all farmland (1019 ha), rising to 64% (1563 ha) by 1896, before slowly declining to a minimum of 696 ha (29%) in 1976. It then recovered to 1018 ha in 2002, approximately its mid-19^th ^century value.

In contrast, temporary grassland only occupied 233 ha in 1866. Amounts remained low (*ca *270 ha) during the later 19^th ^century, declining further, to 86 ha, by 1941. After 1945, the area of temporary grass increased sharply, to 813 ha in 1976, exceeding the area under permanent grass. More recently, areas have fluctuated (1946–2002 mean = 567 ha).

Rough grazing was not recorded until 1891, when it accounted for 32 ha. It then gradually expanded, exceeding temporary grass during the period 1906–1951, peaking at 586 ha in 1936. It then declined, before stabilising at 100–200 ha after 1971. In AD 2000, the last year when it was recorded, rough grazing covered 138 ha.

Cereals occupied 712 ha in 1866, from which they declined to 181 ha in 1936. During the 1960s, hectareage increased sharply, to 693 ha in 1971, exceeding temporary grass, but has since declined slightly (1976–2002 mean = 512 ha). Until the 1950s, wheat, barley and oats were the main cereal crops. From 1961 to 1976 barley was predominant, but by 1981 the area under wheat had recovered, to 326 ha in 2002. Since *ca *1990, maize has increased to an importance equal to that of barley.

Arable land (other than cereals) has declined steadily throughout, from 411 ha in 1870 to 36 ha in 1971, recovering slightly, to 98 ha in 2002. The decline is accounted for by reductions in root crops, beans and peas, field vegetables and potatoes during the late 19th and throughout the 20th century [31]. Set-Aside, only reported after 1990, increased from 50 ha to 199 ha in 2002, an area now exceeding that under arable (98 ha).

### Livestock

**Figure 3:**
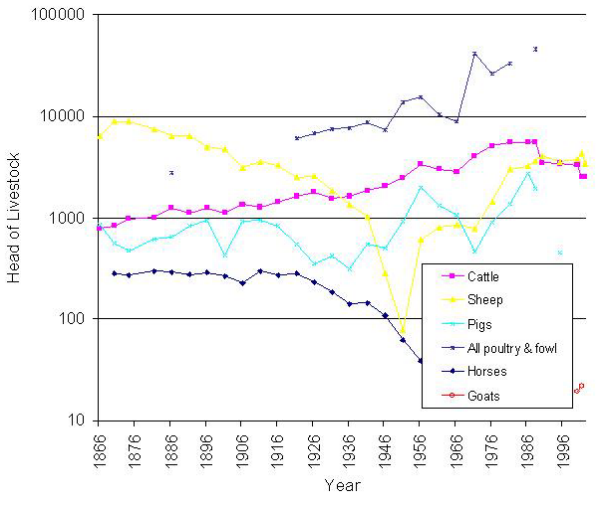
Livestock numbers (head) in the catchment of the Fleet Lagoon, AD 1866–2002.

During the 19th and early 20th century, sheep (Figure 3) were the main livestock in the catchment of the Fleet (excepting poultry). Numbers reached a maximum of 8884 in 1870 (>3 sheep ha^-1 ^of total area reported). They then fell steadily, to only 79 by 1951, but recovered to 3411 in 2002.

Cattle numbers rose steadily from 1866, to a maximum of 5645 in 1988 (>2 head ha-1). Values declined slightly during the 1990s, to 2506 by 2002. Pig numbers fluctuate, with a mean of 647 between 1866 and 1951, and 1407 between 1951 and 1988, in concert with the 'pig cycle'. From 1988, pigs are infrequently reported in the Parish Summaries, and values cannot be determined.

Horse numbers consistently exceeded 200 head until 1926, with a mean for the period 1870–1926 of 274. After 1926, values declined, to 39 in 1956, the last year in which they were recorded. Trends poultry and fowl numbers can only be assessed for the interval 1921–1981, owing to gaps in information.

### Inorganic fertiliser application rates and losses

**Figure 4:**
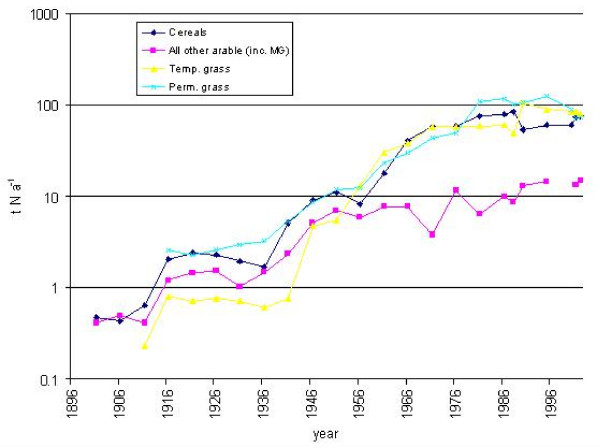
Inorganic nitrogen fertiliser application rates to agricultural crops and grassland (t yr-1) in the catchment of the Fleet Lagoon, AD 1866–2002.

During the later 19^th ^century, inorganic nitrogen fertiliser application rates to crops and grassland (Figure 4) were less than 1 kg N ha-1 yr-1. Applications of inorganic phosphorus (*ca *2 kg P ha-1 yr-1) were approximately double. During the early 20^th ^century, use of both nitrogen- and phosphorus-based inorganic fertilisers increased, with cereals and arable crops receiving the greatest applications.

By 1951, inorganic nitrogen inputs to crops and grassland were greater than those for phosphorus, with all cereals and arable crops receiving 38 kg N ha-1, temporary grass 21 kg, and permanent grass 9 kg. During the same year, inorganic phosphorus applications to cereals and other crops peaked at *ca *28 kg P ha-1 yr-1, before declining gradually to *ca *14 kg for all cereals, and *ca *18 kg for all other crops. Inputs of inorganic phosphorus to temporary and permanent grassland, peaked respectively at *ca *20 kg, and 12 kg P ha-1 yr-1, in 1966 and 1971, before declining steadily to *ca *12.5 kg and *ca *7 kg P ha-1 in 2002.

Inorganic nitrogen inputs to cereals, other crops, and all grasses, increased at a greater rate than those of inorganic phosphorus, until they reached a maximum during the 1980s. Arable land and market gardens generally received the greatest applications, although in 1995, the largest inputs (191 kg ha-1 yr-1) were to temporary grass. Application rates stabilised towards the end of the 20th century, with cereals receiving 145 kg N ha-1 yr-1, all other crops 153 kg, and temporary and permanent grassland 147 kg and 74 kg respectively.

Between 1936 and 1995, the total amount of inorganic nitrogen applied to the catchment of the Fleet as fertiliser exhibits a strong upward trend, from *ca *7 t N yr-1, to *ca *290 t, a more than forty-fold increase (Figure 4). The mean for 1901–36 (Table 4) differs markedly from that for 1981–2002. Losses of inorganic nitrogen from agricultural land, including unfertilised rough grazing, also increase. Mean values for the intervals 1886–1956 and 1981–2002 indicate an approximate fourfold increase between the early and the late 20th century.

**Table 4:** Mean application and loss rates for inorganic and organic nitrogen and phosphorus in the catchment of the Fleet Lagoon, for selected intervals, 1866–2002.

		Nitrogen			Phosphorus		
		
		Interval	(t yr-1)	% increase	Interval	(t yr-1)	% increase
Inorganic	Applied	1901–1936	39.92	641	1866–1936	6.37	439
		1981–2002	256.17		1941–2002	27.99	
	Lost	1901–1936	5.01	442	1866–1936	0.15	387
		1981–2002	22.16		1941–2002	0.58	

Organic	Applied	1866–1966	125.1	213	1866–1936	17.85	190
		1981–2002	266.94		1941–2002	33.39	
	Lost	1886–1956	20.5		1866–1966	0.50	
		1981–2002	43.17		1941–2002	0.95	

**Figure 5:**
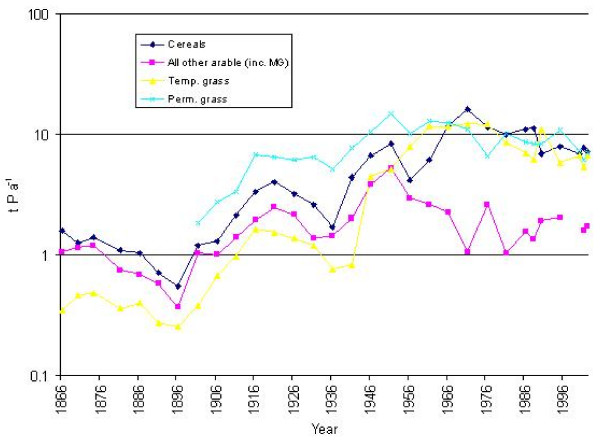
Inorganic phosphorus fertiliser application rates to agricultural crops and grassland (t yr-1) in the catchment of the Fleet Lagoon, AD 1866–2002.

Amounts of inorganic phosphorus (Figure 5) applied as fertiliser during the late 19th century exhibit a slow decline, with a mean of *ca *2.3 t P yr-1 for the interval 1866–1896. Values then fluctuate, with a series of peaks for 1921, 1951, and 1976, when a maximum of *ca *40 t P yr-1 was reached. Applications then fall to *ca *23 t P yr-1 by 2002. Comparison of mean rates for 1866–1936 and 1941–2002 (Table 4) signify growth of 439%. Total losses of inorganic phosphorus follow the same trend, with a 387% increase between the early and the late 20th century.

### Organic nitrogen and phosphorus from livestock

**Figure 6:**
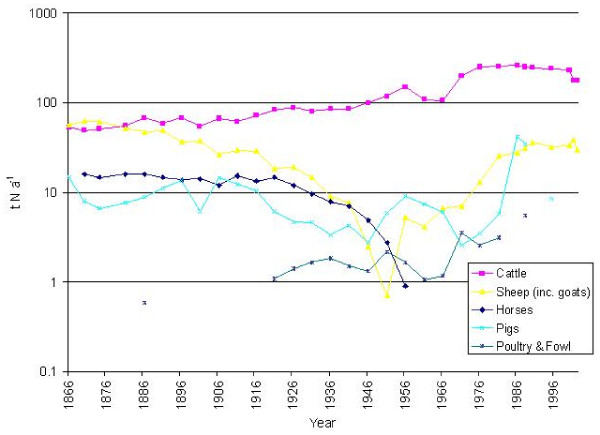
Organic nitrogen production by livestock (t yr-1) in the catchment of the Fleet Lagoon, AD 1866–2002.

The mean value for the total amount of organic nitrogen produced by livestock in the catchment of the Fleet for the period 1866–1966 (Table 4) is *ca *125 t N yr-1, with little discernible change, except for a peak of *ca *166 t N yr-1 in 1956 (Figure 6). Organic nitrogen inputs then grew rapidly between 1966 and 1986, reaching a maximum of *ca *329 t N yr-1, equivalent to *ca *132 kg N ha-1 yr-1. During the 1990s, there was a slight decline, to *ca *206 t N yr-1 in 2002. Total organic nitrogen losses from the catchment increased from 20.5 t N yr-1 for the interval 1866–1966, to *ca *43 t N yr-1 for 1971–2002, with the greatest loss (*ca *53 t N yr-1) in 1986.

**Figure 7:**
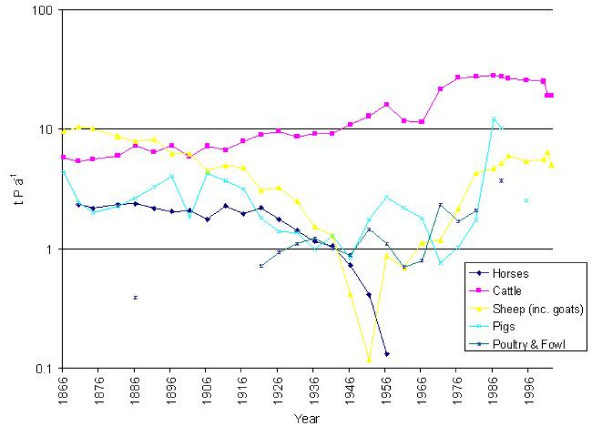
Organic phosphorus production by livestock (t yr-1) in the catchment of the Fleet Lagoon, AD 1866–2002.

Total organic phosphorus produced by livestock in the catchment follows a similar trend (Figure 7), rising from a mean of *ca *18 t P yr-1 in 1866–1966 to *ca *33 t yr-1 in 1971–2002, an increase of 190%. Maximum total production (*ca *47 t, or *ca *19 kg P ha-1 yr-1) occurred in 1988. Values then declined to *ca *24 t P yr-1 in 2002. Mean losses for the period 1866–1966 (Table 4) reach 0.5 t P yr-1, rising to 0.95 t P yr-1 in 1971–2002. The greatest loss (1.3 t P yr-1) is recorded in 1988. Percentage increases for these intervals increase by the same amounts for applications as for losses, as the latter figures are calculated from the former.

### Contributions from wildfowl

Owing to the intermittent nature of the data available, it was not possible to calculate nutrient loadings to the Fleet from wildfowl for each year of the study. Instead, data on current numbers were used to derive a 'flat rate' contribution from this source, which is then employed as a maximum likely input for recent decades. It is thought that numbers are likely to have been lower during earlier times, owing to the absence of modern wildlife conservation measures.

Data on wildfowl numbers on the Fleet for the period 1996–2000 were kindly supplied by Mr D. Wheeler of Abbotsbury Swannery. These figures were then multiplied by the coefficients listed in Table 12 (below) in order to calculate loadings to the Fleet from this source (Table 5). In order to allow for annual migration of some species, and permanent presence of others, mean numbers of 'bird residence days' (BRDs) per year for each wildfowl type were calculated.

**Table 5:** Nitrogen and phosphorus loadings to the Fleet Lagoon from wildfowl.

		Loading (kg yr-1)	
		
	Mean number bird residence days (yr-1)	Nitrogen	Phosphorus
Swans	325284	780.68	149.63
Geese	161246	253.16	790.10
Gulls	418808	184.28	100.51
Other wild fowl & waders	954527	687.26	210.00
Total load (t yr-1)		1.9	0. 54

Annual nutrient loadings were then estimated by multiplying the number of BRDs for each wildfowl type by the appropriate coefficient. Species present on the lagoon for three months per year or less, or with a resident population of fewer than ten birds, were disregarded. Residence was assumed to imply 100% delivery of faecal material to the lagoon, and none to the adjacent land, the 'worst case' scenario also adopted by other modellers [33]. Current loadings to the Fleet from wildfowl, as calculated here, therefore represent just under 2 t nitrogen, and *ca *0.5 t phosphorus, per annum. Of these (assuming a current population of *ca *400 adults [29]), *ca *0.35 t nitrogen, and *ca *0.07 t phosphorus, are supplied by the swans of Abbotsbury Swannery.

### Contributions of nitrogen and phosphorus from sewage

As stated earlier, two small STWs presently discharge into streams draining into the Fleet [12,20,33]. Before these were constructed, it is unlikely that raw sewage was discharged directly into these streams. Instead, material accumulated in earth closets would probably have been disposed of locally (e.g. by burial). Therefore, building these STWs did not reduce sewage inputs to the lagoon, but changed them from non-point to point sources, effectively increasing them. This conclusion is borne out by palaeolimnological and historical studies of other, similar UK water bodies in rural locations [6,8].

Abbotsbury STW became operational in 1966 (email from Wessex Water plc, 2003; email from West Dorset District Council, 2003), serving a local population of 424. The total annual nutrient load discharged to the Fleet that year, as calculated here, was 0.9 t nitrogen, and 0.11 t phosphorus. By 1976, sewered population had fallen to 400, leading to a decline in total sewage nitrogen load to 0.86 t. Sewage phosphorus loads increased gradually, however, to 0.13 t, owing to increased use of phosphorus-based detergents.

Discharges from Langton Herring STW began in 1981 (correspondence from Wessex Water plc, 2002) leading to an increase in sewered population of the catchment of the (West) Fleet of 55%, to 618. Sewage nitrogen load rose in proportion to population (to 1.32 t), whilst phosphorus load almost doubled to 0.21 t. A gradual increase in local population occurred during the 1990s, reaching a maximum of 762 in 2002. Nutrient loads from the two STWs in that year totalled 1.63 t N, and 0.3 t P, an 81%, and a 173% increase, respectively, from 1966.

### Woodland, urban areas, non-agricultural land use, precipitation

On the basis of analysis of maps spanning the period 1870 to the present, total areas of woodland, settlement and non-agricultural land use are assumed to have remained constant throughout. Results for the catchment of the Fleet as a whole are displayed in Table 6. Also shown is total annual nitrogen load from precipitation falling directly upon the surface of the Fleet, plus that which arrives indirectly as run-off from the catchment. Phosphorus loadings from precipitation at this site are considered negligible.

**Table 6:** Total nutrient losses from the catchment of the Fleet Lagoon from woodland, urban and non-agricultural land use, and total nitrogen load received from precipitation.

*Land Use*		Area (ha)	total loss (t yr-1)	
			
			Nitrogen	Phosphorus
Woodland		90.35	1.17	0.002
Urban		78.51	0.39	0.079
Non-agricultural land		44.78	0.58	0.001
Total			2.15	0.081

*Input from Precipitation*				

	Area (ha)	Export Coefficient (kg N ha-1 yr-1)	Run-off Coefficient	Total Load (t yr-1)

Catchment	2807.08	12	0.38	12.8
Lagoon Surface	478.02	12	1.0	5.736
Total				18.537

### Total annual nutrient loadings upon the Fleet lagoon, 1866–2002

#### 1. Nitrogen (Figure 8)

**Figure 8:**
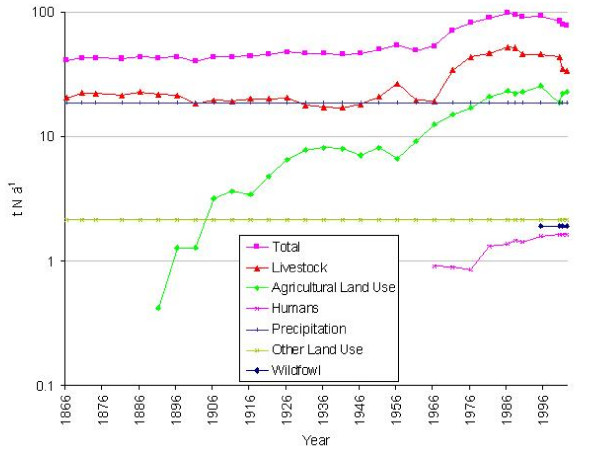
Nitrogen loadings (t yr-1) upon the Fleet lagoon, 1866–2002.

In 1866, the total annual nitrogen load from its catchment upon the Fleet, was *ca *41 t, a value which remained relatively constant throughout the later 19th century. Following a slight decline (in 1901), a gentle upward trend began, culminating in a peak of *ca *54 t in 1956, before the load fell to *ca *49 t in 1961. The mean for 1866–1966 was *ca *45 t N yr-1, but this rose steeply during the 1970s and 1980s, to a maximum of *ca *98 t in 1986, before declining to *ca *79 t in 2002. The increase was driven by a gradual rise in losses from agricultural land in general, but a disproportionate increase in contributions from livestock.

From 1946 to 1986, the greatest part of nitrogen loads upon the Fleet was derived from farmyard manure (FYM), which contributed more than 50% of total load during the 1970s and 1980s, illustrating the significance of increases in livestock numbers. The more recent importance of inorganic fertilisers is demonstrated by a rise in contribution, from approximately 15% in 1946, to more than 20% in 1966.

Percentage contributions of nutrient sources to total load show how precipitation has declined in relative importance, from 45% of total nitrogen in 1866, to 24% in 2002. Livestock were the main source of nitrogen throughout the period 1866–2002, whilst losses from inorganic fertiliser inputs to agricultural land grew in importance, from 3% of total nitrogen in 1896, to a maximum of 24% in 1986. Sewage and other land uses are only minor contributors to total nitrogen load, accounting for *ca *5% in 2002.

#### 2. Phosphorus (Figure 9)

**Figure 9:**
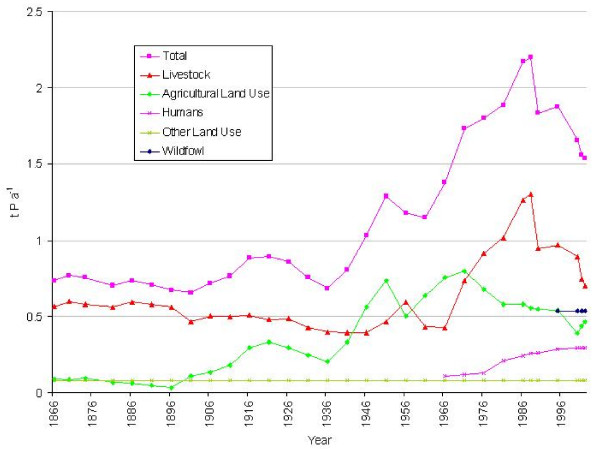
Phosphorus loadings (t yr-1) upon the Fleet lagoon, 1866–2002.

In 1866, the total phosphorus load on the Fleet was *ca *0.75 t, a value which did not change appreciably until the 20th century. Peaks of *ca *0.9 t in 1921, and *ca *1.3 t in 1951, were caused by increasing contributions from inorganic fertilisers (Figure 5). Total phosphorus loadings gradually rose from 1936, to a maximum of 2.2 t in 1988, mainly as a result of growth in livestock numbers, along with increasing contribution from sewage. After 1988 there was a gradual decline, to *ca *1.5t in 2002. Comparison of mean loads for 1866–1936 with those for 1941–2002, (Table 4) indicate expansion from 0.75 to *ca *1.6 t P yr-1, an increase of 104%.

Livestock were the main 19th century source of phosphorus, accounting for ca 80% of total load. From the turn of the century, losses from use of inorganic phosphate fertilisers played an increasing role, from <5% of total load in 1896, to 54% in 1966, before declining during the 1980s. Phosphorus loadings from sewage steadily increased after 1966, from 8% of total load, to 18% in 2002. Phosphorus from other land uses played a minor and diminishing role, declining from 11% of total load in 1866, to 5% in 2002.

## Discussion

### The general agricultural context

Less than 14% of land in the catchment of the Fleet is non-agricultural. Therefore, it is not surprising that the most significant influence upon changing nutrient loads to surface waters since 1866 has been agriculture. Local trends in land use change, cropping practises, and livestock numbers and inorganic fertiliser use, provide a basis for interpreting changes in the trophic status of the Fleet. These changes, especially the strong growth in nitrogen and phosphorus export to standing waters after 1945, may also be viewed in the context of UK national agricultural history.

The first half of the period studied was one of general economic stagnation [34-36], with consequently very little real rise in nutrient inputs from agriculture to the waters of the Fleet. Increases in nitrogen inputs to the lagoon between 1946 and 1986 can then be explained by three local changes which reflect parallel national trends. These are

1. a steady rise in stocking rates of cattle, and recovery of sheep numbers from their minimum 1951, which led to greater amounts of FYM being applied to the land.

2. an increase in land under production, with a decline in the area of rough grazing, and a rise in cereal cropping, leading to intensification of operations.

3. a steep rise in amounts of inorganic nitrogen fertilisers applied to all agricultural land, and in particular to temporary and permanent grass, producing an increase in inputs of inorganic nitrogen.

### Nitrogen loadings

**Figure 10:**
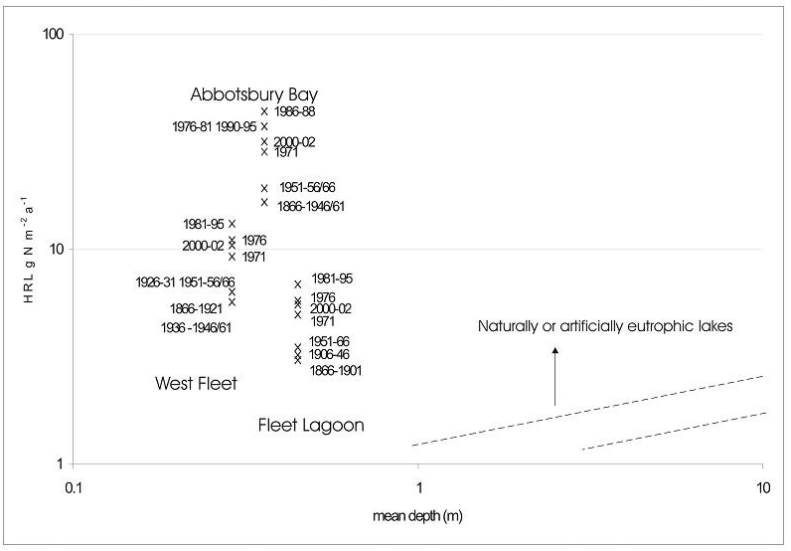
Areal nitrogen loadings (HRL g m^-2 ^[38]) on the Fleet (1866–2002), expressed using Vollenweider's first model [37].

Areal nitrogen loadings on the Fleet for 1866–2002, are expressed using Vollenweider's first model [37], which explores the relationship between nutrient loading and water body mean depth (Figure 10). Values are shown (in the original model) for bodies which are 'oligotrophic' (in this context, not overloaded with nutrients), and those which are naturally or artificially 'eutrophic' (i.e. overloaded by nutrients, in relation to bodies of that area). Owing to the very high nitrogen loadings of the Fleet, only part of the original model is used here. Loadings are shown for (a) the Fleet as a whole, (b) the West Fleet (the much less tidal part of the lagoon), and (c) Abbotsbury Bay, which receives *ca *38% of the total surface drainage (see Site description, above). Rather than gross annual nitrogen loading, Hydrologically Relevant Load (HRL; [38], see Methods, below) is used in order to represent the effective areal loading of the element.

According to this result, the Fleet as a whole has been grossly overloaded with nitrogen throughout the period of study. This is mostly a consequence of its very small mean depth (<0.5 m), along with its very high areal loading (*ca *3 – *ca *7 g m^-2^). Little change occurred until 1945, after which loading increased rapidly (with a slight decline for 1961) until the peak year of 1986, before falling slightly by 2002. In 1946, total annual nitrogen HRL was *ca *48 t, whereas by 1986, it had risen to *ca *100 t, an increase of 104%. This finding clearly illustrates the local effects of the general late 20th century UK agricultural intensification.

By 2002, as a result of declining numbers of cattle, a reduction in the area of cereals and of temporary grass, and a fall in the rate of inorganic fertiliser application to permanent grassland, loads had returned to those of the early 1970s. It is likely that at least some of this reduction is due to the recent implementation (in the Abbotsbury area) by the Ilchester Estate (which owns much of this part of the catchment), of a Countryside Stewardship scheme, a programme designed to reduce intensity of impact of farming on the environment [39]. Non-disclosure of statistics may also be involved, however, with pigs recorded only once between 1988 and 2002. Also, for the period 2000–2002, between 150 and 227 ha were listed as 'unclassified'.

Nitrogen loadings for the West Fleet exhibit similar trends, with little increase between 1866 and 1966. Values for the 1970s reached 10 g m^-2 ^yr-1, and, during the 1980s and 1990s, a peak of *ca *13 g m^-2 ^(1986). By 2002, they had returned to *ca *10 g m^-2 ^yr-1. Loadings for Abbotsbury Bay are very high indeed, varying between *ca *16 and 21 g m^-2 ^yr-1 for 1866–1966, rising to *ca *28 g m^-2 ^by 1971, and then *ca *36 – ca 44 g m^-2 ^yr-1 for the late 20th century. Values have now (2002) fallen somewhat, close to those of the early 1970s.

### Phosphorus loadings

**Figure 11:**
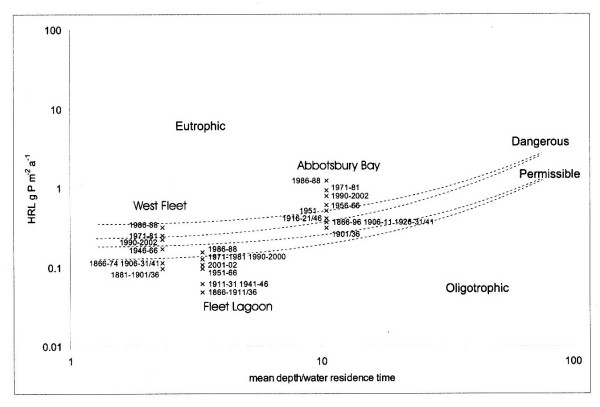
Areal phosphorus loadings (HRL g m^-2 ^[38]) on the Fleet (1866–2002), expressed using Vollenweider's second model [40].

Vollenweider's second model [40] predicts the trophic status of a water body from mean areal phosphorus loading versus mean depth, divided by water residence time (Figure 11). Values for the last for various parts of the Fleet are estimated from [14] and [15], but may only be approximate. Loadings are defined in terms of critical limits [38,40], these being (a) 'permissible' – that loading unlikely to force oligotrophic bodies to change from their present state, and (b) 'dangerous' – that likely to continue to force eutrophic bodies to change [41]. Again, rather than total annual load, HRL [38] of phosphorus is used here in order to calculate critical loadings of the element.

Phosphorus loadings for the Fleet as a whole remained within permissible limits throughout the period 1866–1981, only exceeding that value during the interval 1986–1988. As with nitrogen, the last decade (1990–2002) has seen total phosphorus loads decline slightly, approximately to those of the 1970s. Loadings on the West Fleet remained higher than those on the Fleet as a whole, but stayed within permissible limits, until the 1940s. They then increased consistently, reaching a maximum, close to dangerous, during the late 1980s. During the last decade they have fallen, but still remain above permissible limits.

Abbotsbury Bay has consistently received the greatest areal loadings of both nitrogen and phosphorus. Phosphorus loads upon the Bay were already greater than permissible by 1866, and exceeded dangerous during the 1950s, again reaching a maximum during the 1980s, with a decline during subsequent decades. Phosphorus loadings on Abbotsbury Bay would need to be reduced to those of the 1950s in order to fall to dangerous values, and to those of the early C20th even to begin to approach permissible limits.

Phosphorus loads on the Fleet first rose as inorganic fertiliser applications reached a peak during the early 20th century. The most significant increases, however, occurred between 1946 and 1988, when annual HRLs rose from *ca *0.5 t yr-1 to *ca *0.9 t, an increase of 175%. This rise is explained by two changes in local agricultural practice,

1. an increase in inorganic fertiliser applications to all crops, and grassland, which peaked during 1951, and again in 1971, and

2. a sustained increase numbers of sheep and cattle, and fluctuating pig numbers, which generated peaks of organic phosphorus applied as FYM in 1956, and in 1988.

Together, these changes led to an initial post-1945 increase in phosphorus loading from inorganic fertilisers, and then a later, separate rise in organic inputs from livestock. FYM is the main source of phosphorus to the Fleet in nearly all years of the study, exceeded by the contribution of inorganic fertilisers only during the intervals 1946–1951, and 1961–1971, when application rates rose to maximum values.

After 1988, total phosphorus loads declined, mainly as a result of a reduction in cattle numbers, a contraction in the area under cereals, and a fall in rates of inorganic fertiliser applications to these crops. Again, this change may at least be partly due to implementation of the Countryside Stewardship scheme at the Abbotsbury end of the catchment. Phosphorus loadings on the Fleet as a whole have returned almost within permissible limits, but not quite. As with nitrogen, non-disclosure of pig numbers, and omission of a small proportion of agricultural land from official statistics, may play a part in reducing the modelled total phosphorus load.

The contribution of sewage effluent to total phosphorus load has become increasingly significant since Abbotsbury STW opened in 1966. By 2002, human load on the Fleet had risen by 165% (from *ca *0.1 to *ca *0.3 t yr-1), contributing approximately 14% of total load. This increase was due mainly to three changes, namely

(a) a marginal rise in local population,

(b) growth in use of phosphorus-based detergents, and

(c) expansion of sewered population, with the commissioning of Langton Herring STW in 1981.

Although human load is small in relation to that from agriculture, it became critical during the years 1986–1988, when permissible limits for areal loading on the Fleet as a whole were exceeded.

As indicated, nitrogen contributions to the Fleet from wildfowl are not particularly important. For phosphorus, however, wildfowl currently contribute a significant proportion of the total budget of the Fleet (*ca *35%, or *ca *7 × that from sewage effluent). Of this, Abbotsbury Swannery currently produces *ca *4.5% of the total phosphorus budget, which is broadly equivalent to the present human input.

The Swannery is therefore currently probably not a major source of nutrient loadings to the lagoon, and historically, its contribution may have been less. One *caveat*, however, is that introduction of increased phosphorus inputs (both from agriculture, STWs and increased numbers of swans) into the relatively confined waters of Abbotsbury Bay, may be responsible for some of the ecological changes recently observed (e.g. decline of eelgrass [21]). The very short water residence time of the Bay (*ca *12.5 days) helps counteract such effects. The bird populations of the Fleet as a whole, are clearly an important source of phosphorus, which for an aquatic site of such great nature conservation value, is not particularly surprising. Unlike sewage effluent, they are also, especially the Abbotsbury swans, part of the inherent value of the Fleet [13], and hence its attraction to human visitors.

Differences in loadings between the three sections of the Fleet, can be explained in terms of catchment hydrology, location of point sources, and morphometry of the lagoon. Approximately 1000 ha (38% of the catchment) drains into Abbotsbury Bay, which itself covers only 28 ha (6% of the Lagoon). Hence, in areal terms, the Bay receives by far the greatest annual loadings of any part of the Fleet. Inputs from Abbotsbury STW (the larger of the two discharging into the Fleet) swell this load further, particularly in terms of phosphorus. The narrowness of the catchment from Abbotsbury to the inlet at Smallmouth (Figure 1) produces lower areal loadings from diffuse sources for the West Fleet and Fleet as a whole. Variations in bathymetry along the Fleet, and the weak influence of tidal flushing compared to that at Smallmouth, indicate that loadings upon Abbotsbury Bay are likely to be the most critical, owing to the attenuated influence of the tidal cycle at the western end of the lagoon.

### Comparison with other studies

Few previous hindcasting studies have been carried out for the Fleet [42,43]. Mainstone & Parr [33] used export coefficients in order to model nutrient loads for 1997. Like us, they concluded that agriculture is the main source of nutrients for the Fleet, accounting for >80% of total annual nitrogen, and 56–69% of total annual phosphorus load. Equivalent values from our study (for 2000), are (respectively) 74% and 77%. Although not directly comparable, owing to differences in methods and data sets used, these estimates are broadly similar. Loadings for 2003, calculated on the basis of a detailed land use survey by Hudson [32], are ca 84–98 t nitrogen, and ca 4 t phosphorus.

Historical land use changes in the catchment of the Fleet are similar to those recorded by Johnes *et al*. [44] in their hindcasting study of ten British river catchments, and also those described by O'Sullivan [6,8,45] in his investigations of Slapton Ley (Devon), Loe Pool (Cornwall) and Bosherston Lakes (Dyfed). Here, intensification after 1945 led to increases in livestock numbers, increased use of inorganic fertilisers, and removal of rough grazing. However, at all sites, there was also evidence of an earlier, late 19th, early 20th century agricultural intensification, and consequent eutrophication, associated with completion of the UK branch line railway network, and connection of previously very rural locations to the national market, particularly in dairy products.

In the data for the Fleet, there is less evidence of this 'railway effect', even though a local branch line did indeed reach Abbotsbury in 1885 [46]. However, the main line to nearby Weymouth (Figure 1), with several local stations located close to the eastern edge of the catchment of the Fleet, had already been built by 1857, so that, by 1866, the area draining into the Fleet had already been connected to the national market. Any local 'railway effect' may therefore precede the beginning of collection of the data we have analysed here.

Overall, our results suggest that, even before 1866, the Fleet was already grossly overloaded with nitrogen, and that, in Abbotsbury Bay, phosphorus loadings had already exceeded permissible levels. Tithe maps and apportionments in the Dorset Record Office [42,43] and records of the Ilchester Estate [28], offer important documentary evidence of earlier historical agricultural practices and land use within the catchment of the Fleet, which could be used to hindcast nutrient loads further.

### Evaluation of restoration strategies

**Figure 12:**
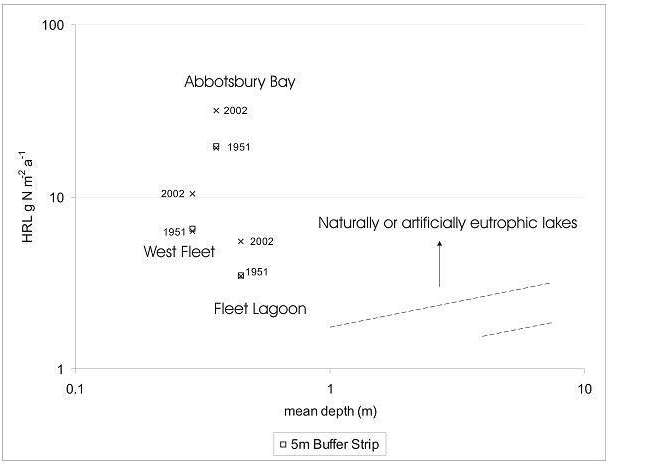
Effects of installation of 5 m riparian buffer strips in its catchment, on nitrogen loadings on the Fleet lagoon, estimated using Vollenweider's first [37] model.

**Figure 13:**
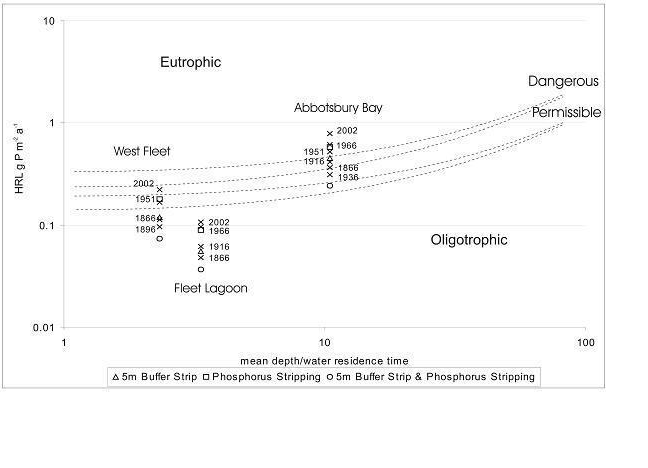
Effects of installation of 5 m riparian buffer strips in its catchment, and of phosphorus stripping at Abbotsbury and Langton Herring STWs, on phosphorus loadings on the Fleet lagoon, estimated using Vollenweider's second [40] model.

Vollenweider's first and second models [37,40] may also be used in order to evaluate proposed strategies for alleviation of eutrophication [6,8]. Here, three measures designed to mitigate nutrient loads upon the Fleet are modelled (Figures 12, 13; Table 7), namely

**Table 7:** Effects of mitigation measures upon nutrient loads upon the Fleet Lagoon.

	Load (t), (% reduction)			
	
	Current load (2002)	With phosphorus-stripping	With 5 m buffer strips	5 m buffer strips & phosphorus-stripping
***Fleet Lagoon***				
Nitrogen	78.68		50.16 (36%)	50.16 (36%)
Phosphorus	1.54	1.28 (27%)	0.79 (49%)	0.52 (66%)

***West Fleet***				
Nitrogen	61.09		38.23 (37%)	38.23 (37%)
Phosphorus	1.31	1.04 (20%)	0.70 (47%)	0.43 (77%)

***Abbotsbury Bay***				
Nitrogen	27.17		16.87 (38%)	16.87 (38%)
Phosphorus	0.67	0.49 (27%)	0.38 (43%)	0.21 (69%)

Percentages express reductions in load compared to base year (2002).

(a) implementation of 5 m riparian buffer strips along all watercourses in its catchment, and around the perimeter of the lagoon,

(b) introduction of phosphorus-stripping at both STWs discharging into the Fleet, and

(c) both [31].

The first measure focuses on diffuse loads of both nitrogen and phosphorus; those which are mainly delivered to surface waters in particulate form during winter, by increased runoff. The second is aimed at lowering phosphorus loads from point sources (i.e. STWs). These are delivered mainly in soluble form, and at constant rates throughout the year (with a possible increase in this case for the tourist season), and therefore tend to reach peak concentrations in inflowing rivers during the growth season. Both kinds of source are important, however, for estimating loadings to standing waters [33,47], where nutrients delivered in particulate form are stored as sediments, and eventually become available for growth via resuspension and remobilisation. Values for buffer strip efficiency were taken from [48] for nitrogen, and [49] for phosphorus. The retention rate of phosphorus stripping at STWs is that used by Johnes [50].

Modelling of results for introduction of 5 m buffer strips for nitrogen (Figure 12) shows that it would be possible to reduce current (2002) non-point loadings upon the Fleet (*ca *79 t) approximately to those of 1951 (*ca *50 t), a fall of 37%. This is a considerable reduction, but would still leave the lagoon grossly overloaded with the element, emphasising the magnitude of increases in nitrogen loadings since 1945. Reductions on the West Fleet, and on Abbotsbury Bay, are of the same order (36–38%). Unlike his second model [40], Vollenweider's first [37] does not take residence time into account, but even when we include this variable in our calculations, we find that diffuse nitrogen inputs to each part of the lagoon continue to exceed dangerous loadings.

The effect of introduction of 5 m buffer strips on diffuse inputs of phosphorus (Figure 13) is greater (43–49% decrease), with loadings on the Fleet as a whole, and on the West Fleet, approaching those of 1866, leading to reduction of inputs to the West Fleet below permissible loading. In Abbotsbury Bay, the effect is attenuated, owing to the residual influence of current sewage inputs, which would prevent reduction of loadings below those of 1916, a value still beyond the dangerous limit for this part of the Fleet.

Installation of phosphorus stripping at Abbotsbury and Langton Herring STWs (Figure 13) would lead to a 27% reduction in point source inputs to the Lagoon (Table 7), to *ca *1.3 t yr-1, a loading equivalent to that of 1951. As loadings of this element to the Fleet as a whole already lie within permissible limits, such reduction would be beneficial, but not critical. For the West Fleet, the equivalent reduction is 20% (to *ca *1 t yr-1), which would still leave loadings on this part of the lagoon just beyond permissible limits. For Abbotsbury Bay, as calculated here, the reduction achieved is also 27%, but marginal effects are much less, with inputs to this part of the lagoon remaining beyond dangerous on the basis of phosphorus stripping alone. Phosphorus stripping may still be an important option to consider for installation at Abbotsbury STW, however, in that the works may have been operating 'above consent' for some years [20].

Joint implementation of both phosphorus stripping and establishment of 5 m buffer strips (Figure 13) would achieve further reductions in phosphorus loadings (66–77%) on all parts of the lagoon, and would serve to reduce inputs to the West Fleet to values further within permissible limits. However, in the case of Abbotsbury Bay, loadings would still lie beyond permissible limits. Such measures might still be worth considering, as the extent of any further reductions in phosphorus loading required, in order to bring this part of the lagoon within permissible limits (e.g. a voluntary ban on use of phosphate detergents), would be quite modest. Even greater reductions could be achieved, for example, by diversion of all sewage inputs from the Abbotsbury end of the Fleet, and further 'extensification' of agriculture.

## Conclusion

Hindcasting studies show that the Fleet lagoon, despite its high conservation status, has been overloaded with nitrogen, throughout the past 140 years. The main source of this element, over the entire period studied, has been agriculture, with sewage effluent and other sources, including the Abbotsbury Swannery, playing only a minor role. Recent agricultural 'extensification' has served to alleviate this condition somewhat, but few measures seem available which might realistically reduce nitrogen loadings. Such a policy might not in any case be entirely desirable, in that the lagoon has clearly retained its great inherent ecological value in the continued presence of such elevated loadings.

Phosphorus loadings could, however, be reduced, for the West Fleet via further agricultural 'extensification', and for Abbotsbury Bay by a combination of this measure with phosphorus removal at the relevant STWs, or even diversion of effluent. As it is highly possible that certain changes in the ecology of the Fleet, particularly those seen recently in Abbotsbury Bay, may be due to 20th century increases in phosphorus loading [12,20], this might be a wise and relatively inexpensive precaution.

## Methods

These are the same as employed in a number of earlier publications [4,6,8-10,44,45,50,51], as originally adapted from Jørgensen [52].

### Historical data

Data on agricultural land use and livestock numbers for the catchment of the Fleet for the period 1866–1988 were compiled using annual Parish Summaries of the June Agricultural Census (England and Wales) located at the Public Record Office, Kew, London. Nine civil parishes, records from which were used to develop data on land use history, lie partly or mainly within the catchment (Table 8). Of these, Abbotsbury, Langton Herring and Fleet make up more than 70% of its total area.

**Table 8:** Civil parishes within the catchment of the Fleet (from [31]).

Civil parish	Area (ha)	Area within catchment (ha)	% parish area within catchment
Abbotsbury	1819.31	1309.04	71.95
Portesham	1825.40	415.96	22.79
Little Bredy	653.04	5.38	0.82
Long Bredy	1402.46	2.49	0.18
Langton Herring	372.11	367.67	98.81
Fleet	348.61	335.92	96.36
Chickerell	1378.18	284.15	20.62
Wyke Regis	2967.32	38.25	1.29
Portland	1206.44	48.23	4.00
Total		2807.08	

Information was collected at five-yearly intervals, adapted when necessary owing to gaps in the records. From 1989, the Census is subject to strict rules of disclosure. Changes in methods of data collection and presentation [53] mean that Summaries are not available for the period after 1988. Alternative sources are DEFRA wards (which usually contain a number of civil parishes), or those developed for Environment Agency (EA) catchment planning (email from DEFRA, 2003).

Using the Agricultural Census also creates a number of interpolation problems [9,54], mainly because, at least in lowland regions, parish boundaries do not always coincide with catchments. Because agricultural holdings also often extend across catchment boundaries, statistics refer to areas which may not exactly coincide with parishes [55,56]. The smaller the proportion of the parish for which data are to be interpolated, the larger the uncertainty [54]. Therefore, here, only those parishes more than 10% of whose total area lies within the catchment were used in order to derive land use and livestock estimates.

### Catchment model

The catchment model employed is that of Jørgensen [52], as adapted by [51,50] and [44]. Estimates of external nutrient loads upon a waterbody are calculated by summing outputs from all potential diffuse and point sources of nitrogen and phosphorus within and over the catchment, as in

(a) natural (i.e. diffuse/non-point) losses

n

LP = Σ·A × EP (where EP = Ep_f _+ Ep_ag _+ EP_u_)

i = 1

where LP = loss of phosphorus (kg ha-1 yr-1)

A = area of watershed (ha)

EP = export coefficient for phosphorus, where:

_f _= forest, _ag _= agricultural land, _u _= urban areas

(b) direct input from precipitation

IP = CP × A_0_

where IP = input of phosphorus

CP = P concentration in precipitation (mg l^-1^)

A_0 _= area of water body (ha)

(c) load from sewage

LP = (D_ca_P × H × 365 × M × B × R_S_)/10^6^

where LP = phosphorus loss (t a^-^')

D ca = phosphorus discharge person^-1 ^(g day^-^')

H = sewered human population

M = loss during mechanical treatment (10–15%)

B = removal during biological treatment (10–15%)

C = removal during chemical treatment (80–90%)

Rs = retention coefficient of filter bed (1–88%)

Here, the original model has been modified in three ways. First, estimated contributions of animal wastes, and of organic and inorganic fertilisers to nutrient loads exported from agricultural land, are quantified separately. Second, agricultural land is not treated as an homogenous unit. Instead, agricultural land use types are differentiated, reflecting variations in predicted rates of nutrient application rates, and hence of nutrient export. These modifications were initiated by [51], and further developed by [50] and [44]. A third modification is that we have introduced coefficients for inputs from wildfowl (including waterfowl), in order to take into account the importance of these populations for the Fleet.

### Inorganic fertiliser application rates and export coefficients

Amounts of inorganic fertiliser applied to agricultural land use types within the catchment of the Fleet were calculated using data from four principal sources. These are: -

1. National average crop applications for the period 1976–2002 from the British Survey of Fertiliser Practice (BSFP; [57]). Between 1942 and 1991, these were compiled by Rothamsted Experimental Station, Harpenden, Hertfordshire, and from 1992 until 1998, by the University of Edinburgh Data Library. In 1999 they became the responsibility of the Rural Business Unit of the University of Cambridge.

2. A report on fertiliser practice in England and Wales presented to the "Closed" Conference of Advisory Soil Scientists Fertiliser and Lime Committee by Church & Webber [58], containing trends for major land use types for the period 1957–69.

3. National trends in consumption of total inorganic nitrogen and phosphorus fertilisers for the period 1913–1956, published in Fertiliser Statistics of the Fertiliser Manufacturers Association [FMA; [59]].

4. National trends in artificial fertiliser use for the period 1851–1956, as derived by Brassley [60].

The BSFP uses a stratified sampling procedure in order to produce regional and national statistics for fertiliser application rates. Owing to lack of regional data for West Dorset, application rates of nitrogen and phosphorus fertilisers used here are based on national figures. Mean application rates for all cereals, all arable (including bare fallow and market gardening), temporary grass and permanent grass, were derived from BSFP national statistics for individual crop types and land uses for the years 1976–2002. Church & Webber [58] did not differentiate between cereals and arable, however, so their summary statistics refer to all tillage. For the period before 1976, it was not possible to derive separate cereal and arable application rates. National trends in inorganic fertiliser use reported by [59] and [60], were used in order to extrapolate application rates for the years 1866–1951.

Where values fall below 1 kg ha-1 yr-1, applications were considered to be nominal, and figures for previous years not calculated. All application rates are given as total nitrogen (kg N ha-1 yr-1), and total phosphorus (kg P ha-1 yr-1). Total inorganic phosphorus applied was calculated assuming that 1 kg of inorganic fertiliser phosphorus pentoxide (P_2_O_5_), contains 0.4327 kg of phosphorus. Appropriate export coefficients for losses of inorganic fertiliser from agricultural land (Table 9) were selected from [4,51,49,44] and [54].

**Table 9:** Export coefficients for agricultural and other land use in the catchment of the Fleet Lagoon (after [4], [44], [50], [51] and [54]).

Agricultural use	Inorganic nitrogen fertiliser application rate (kg N ha-1 yr-1)	Nitrogen	Phosphorus
		
		%	
All cereals		12	2.5
Arable	>120	30	4.2
	60–120	20	
	30–60	7	
	<30	3	
Temporary grass		5	1.5
Permanent grass		5	1.5
Market Gardening		20	4.2

		kg ha-1 yr-1	

Rough grazing		13	
Setaside			2
All other agricultural land		n.a.	
Undisclosed crops/land use			

Loss coefficients for agricultural land are based on % of total amount of fertiliser applied, but on absolute rates for other land use types.

### Organic fertiliser application rates and export coefficients

Annual amounts of nitrogen and phosphorus produced by livestock in the catchment of the Fleet were calculated using figures derived by [51] from a comprehensive review of literature sources (Table 10). Goats – not included in their study – were assumed equivalent to sheep [37]. Unlike inorganic fertilisers, animal waste is not applied in total to the land. Instead, some is voided directly whilst animals are grazing, whereas that dropped in stock houses is collected, stored, and applied as farmyard manure (FYM) or slurry. Coefficients derived by [50] are therefore calibrated in order to account for losses of volatile forms of nitrogen (mainly ammonia) during storage, and uptake by crops.

**Table 10:** Nitrogen and phosphorus production by livestock, and percentage losses in run-off (based on [51]).

	Characteristic loadings ^†^ (kg ca-1 yr-1)		% of manure voided:				% of manure voided lost to run-off	
			
	N	P	directly to land	in stock houses and stored	% lost during storage	% of total applied to land	N	P
Cattle	70.2	7.65	50	50	10	95	16.15	2.85
Sheep	8.9	1.5	100	0	0	100	17	3
Pigs	18.75	5.63	0	100	15	85	14.45	2.55
Poultry	0.3	0.2	0	100	10	90	15.3	2.7
Horses	76.8	11.4	50	50	10	95	16.15	2.85

^† ^kg ca-1 yr-1 = kg per *capita *per *annum*.

### Non-agricultural land use

Areas under non-agricultural use (*i.e*. woodland, urban areas, all other major uses) were calculated using GIS from Ordnance Survey Explorer™ map OL15 [61]. Inspection of earlier maps held at the Dorset Record Office, and in Morris [28], indicates that areas devoted to these land uses have remained constant for the period of study, except in very limited parts of the catchment at the southern end, close to Weymouth (Figure 1). Appropriate export coefficients for these uses (Table 11) were selected from those derived by [51,50,44] and [54].

**Table 11:** Export coefficients for non-agricultural land use (based on [44], [50], [51] and [54]).

Land use type	Nitrogen loss (kg ha-1 yr-1)	Phosphorus loss (kg ha-1 yr-1)
Urban	5.0	1.0
Woodland	13.0	0.02
Other major uses	13.0	0.02

### Wildfowl

Export coefficients for the main types of wildfowl present on the Fleet, based mainly on values given by [62] and [63], are shown in Table 12. No values for Mute Swans could be found, so that coefficients are based on those given by [64] for Black Swans (*Cygnus atratus *Latham; 1.2 g N and 0.23 g P day^-1^), multiplied by two, in order to account for the greater body weight of Mute Swans.

### Point sources

Locations were identified from the same base map as non-agricultural land use. Dates for when they came into operational use, and the treatment provided, were obtained from West Dorset District Council, and Wessex Water plc. Total sewered population was estimated from parish population figures obtained from the UK National Population Census, adjusted according to percentage of the parish falling within the catchment of each STW. Characteristic loadings of nitrogen and phosphorus (kg ca-1 yr-1) from domestic sewage waste (Table 13) are those used by [4,51,49], and [44]. Coefficients for nitrogen and phosphorus removal during sewage treatment are based on those derived by [50].

**Table 12:** Export coefficients for the main types of wildfowl present on the Fleet Lagoon (based on values given by [62], [63] and [64]).

	g ca-1 day-1	
	
	Nitrogen	Phosphorus
Swans	2.4	0.46
Geese	1.57	0.49
Gulls	0.44	0.24
Other wild fowl & waders	0.72	0.22

**Table 13:** Nitrogen and phosphorus loads (kg ca-1 yr-1) produced by domestic sources, and coefficients for removal during sewage treatment (based on [44], [50] and [51]).

Source (kg ca-1 yr-1)	Nitrogen	Phosphorus
Human Load	3.94	0.65
Detergent Load (base year 1988)		0.51
Removal during secondary treatment (%)	n.a	67.22
Total sewage load after secondary treatment†	2.14	0.38

^† ^based on an original [49] figure of 10.8 g N ca-1 yr-1, equal to an annual loading of 3.942 kg N ca-1 yr-1, receiving primary and secondary treatment as per [48], with retention coefficients of 15% for mechanical treatment, 20% for biological, and 20% for retention by filter beds.

The quantity contributed to sewage *per capita *from phosphorus-based detergents, was adjusted according to national trends in consumption in the *Monthly Digest of Statistics *[65], an approach originally developed by [4]. This was only possible for the period 1966–1990, owing to changes in the statistical method employed in order to determine national trends from gross sales. The contribution of detergents to UK sewage phosphorus loads increased by 69% between 1961 and 1990, from 0.77 to 1.29 kg P ca-1 yr-1.

### Marine inputs

Previous modelling studies of the Fleet [12,20,33,66] regard nutrient inputs from marine sources as minor compared to loadings from its catchment. Existence of a strong gradient in nutrient concentrations from Abbotsbury (the northwestern, landward end of the lagoon; Figure 1) to Smallmouth (the entrance), which varies directly opposite to that of salinity, also strongly suggests that catchment nutrient sources are much more significant than marine inputs. It is possible [20] that Portland Harbour does constitute a source of nitrogen (as nitrate), and a small source of phosphorus (as orthophosphate) to the Fleet, but on the flood tide, and only to the East Fleet, where tidal flushing is much stronger, and not to the much more highly nutrient-rich West Fleet. It is also not clear whether this process represents a net loss or gain [20].

### Trophic classification

The trophic state of the Fleet Lagoon since 1866 was predicted at five-year intervals using Vollenweider's first model [37] for nitrogen, and his second [40], for phosphorus. Rather than total annual load, calculations are based on hydrologically relevant load (HRL) i.e. that proportion of the total annual load entering a waterbody during the growth season [38]. This was approximated to one third of total annual load, for both nitrogen and phosphorus [4,6]. Areal nitrogen and phosphorus loadings (g m^-2 ^yr-1) were calculated (a) for the Fleet as a whole, (b) for the West Fleet (as defined in Figure 1), and (c) for Abbotsbury Bay. Values for mean depth, water residence time, and tidal flushing rate were determined from the model developed by Robinson [14,15].

## Abbreviations

AD – calendar year *Anno Domini*

BRDs – Bird Residence Day (for explanation, see text)

BSFP – British Survey of Fertiliser Practice

DEFRA – (UK) Department of Environment, Food and Rural Affairs

EA – Environment Agency (England and Wales)

EC – European Community

EEC – European Economic Community

EU – European Union

FMA – Fertiliser Manufacturers Association

FYM – Farm Yard Manure

HRL – Hydrologically Relevant Load

n.a. – not applicable

Plc – Public Liability Company

SAC – Special Area of Conservation

SSSI – Site of Special Scientific Interest

STW – Sewage Treatment Works

TON – Total oxidisable nitrogen

UK – United Kingdom of Great Britain and Northern Ireland

## Competing interests

The authors declare that they possess no competing interests, and also that the opinions expressed in the paper are their own, and not those of the Environment Agency.

## Authors' contributions

GW collected the majority of the data, and carried out most of the modelling calculations and drafted the text. PO'S supplied some data, oversaw the modelling exercise, advised regarding export coefficients, and final edited the text. PB supplied historical information, and advised on export coefficients for earlier parts of the period investigated. All three authors read and approved the final text.
